# Traveling Letters, No. IV

**Published:** 1844-09

**Authors:** 


### THE WESTERN JOURNAL.

NEW SERIES.—VOL. II.—NO. III.

LOUISVILLE, SEPTEMBER 2, 1844.

### TRAVELING LETTERS FROM THE SENIOR EDITOR.

NO. IV.

KANZAS RIVER, 'Indian Territory,'
August, 1844.

*To my Colleagues:*

GENTLEMEN:—I find that the time for dispatching a letter for our September number, has overtaken me at a place that will not admit of my giving you 'some account' of the far and ill-famed 'Black Tongue,' as I had intended to do; for as I passed up through St. Louis, unwilling to trust my southern notes to the treacherous Missouri, I deposited them in that city. I hope to return to them in time to give you a notice of that epidemic Erysipelas, for our October Journal. Meanwhile, I trust that some of our brethren who have encountered it, will send you the result of their experience.

Called upon to write from the FAR WEST, it would seem appropriate for me to say something on what immediately surrounds me, and I shall begin by giving you a page or two on

*The Indians.*

The tribes and fragments of tribes, which are found in this quarter, are the Shawanoese on the south side of the Kanzas, (or as it is here familiarly called, the *Kau* river), and the Delawares, Wyandotts, Kickapoos, and Pottawatamies on the north, extending up the Missouri to a few miles above Fort Leavenworth. They are all more or less civilized, and have missionaries, teachers, and traders residing among them. As the game in this quarter is nearly destroyed they must work or starve. You might suppose that such a dilemma would spur them into active effort, but as it is a difficult matter to starve an Indian, not a few of them are as idle as a young doctor in the first year of his probation. They have, however, the advantage of him in this, that they look forward to a *certain*, though small, annual stipend from the Government, while he has no positive assurance of a good fee, when his year of waiting has expired. In another aspect of the matter, they are in much the same predicament—when the year has rolled round the debts of both generally swallow up their receipts. But as these remarks are by no means applicable to all our junior brethren, so they must not be applied to all our red brethren; for some of them are really industrious and thrifty. I visited a Kickapoo, who was comfortably lodged, had his plantation divided off with good fences into fields for corn, wheat, meadow, *truck* and, in imitation of a Missouri farmer, even hemp; while his lands and yards were well filled with cattle, horses, and hogs, which in the evening had returned from the neighboring woods and prairies, and made quite a show in [illegible] lane.

On examining the heads of three small children of this agricultural nabob, *Char-kee-ka*, (his name deserves to be recorded), I found them all flattened in the occipital region, so that they had lost the carinate and oval form, and assumed a cylindrical. This, the Indians present ascribed to the binding of them to a board, immediately after birth. The head is not bound, it is true, but as the board lies down, the weight of that extremity rests constantly on the occiput. The heads of most of the several adults present, presented something of the same compression; but when I went into another tribe, the Shawanoese, I found it much less. On the Kickapoo theory of this modification of the cranium, I shall not stop to offer any criticisms, and if either of you should kick up at it, I shall expect you to propose a better.

During the same afternoon, I saw two cases of half-formed cataract, two of pterygium, and, subsequently, one of strabismus, all in women and girls. A 'fine chance' for a young ophthalmic surgeon, *vulgo*, an oculist! I hope some of Professor Gross' pupils will find them out, especially the last, for an Indian girl has not quite beauty enough to afford to squint. By the way, the female offspring of the red and white varieties of our race, are not as comely as mulattoes and quart'roons, especially those of the Gallic mixture. In the compound of the Indian and Anglo-Saxon or Gallic blood, I have here abundant evidence, that the dark color of the iris outlives that of the hair, as I meet with many examples of black eyes, with chestnut and even sandy hair.

The most prevalent Indian disease is scrofula. This is the testimony of those who have spent years among them. It has almost utterly extirpated the fragmentary tribe of Peorias, living on the sources of the Osage river. They generally died with cough and expectoration; but in many, it is limited to the lymphatic ganglia of the neck. In different schools and families I have had an opportunity of examining about 150 children, and find at least 12½ per cent., more or less, affected with that disease. While I was at the admirable Manual Labor School of the Methodist Episcopal Church, a Kanzas boy, laboring under pulmonary scrofula, suddenly expired of hæmoptysis. At the Baptist mission, among the Shawanoese, I saw a man with scrofula of the skin of his neck and breast, and in various places I have seen the eschars left after spontaneous cures.

At the risk of disappointing such of our readers as have no relish for any thing but medicine, I cannot refrain from saying a few words on the efforts making in this quarter to raise the tribes into a civilized condition. The Baptist Missionaries have chiefly aimed at the direct introduction of christianity; the Society of Friends, mainly at education in letters and the domestic arts; the Methodists at the whole, not successively, but simultaneously. It is impossible for me to doubt that this is the true method; and, while I cheerfully accord to all these denominations of christians, an equal degree of liberality, zeal, and self-denial, I am compelled to prefer the system of the latter. Till lately, they had four tribe schools, among the Peorias, Shawanoese, Delawares and Kickapoos, one each. They have abolished the whole, and formed one institution, a few miles south of the Kanzas river in the edge of a beautiful grove, nourished by half a dozen springs. Of an adjoining fertile and rolling prairie, they have enclosed 500 acres, three-fourths of which are cultivated by the labor of hired Indians, while the remainder is overspread with domestic animals. Several trades, including the manufacture of meal and flour by steam, are here carried on, to all of which Indian men or boys are parties; and show, as I witnessed, an encouraging degree of ingenuity and diligence. But the most interesting part of this establishment is the school, which embraces nearly 100 children and young persons, about two-fifths of whom are girls. In addition to spelling and reading, they learn writing, in which they make rapid progress, arithmetic, and music by note, evincing a talent for this latter accomplishment, much greater than has been ascribed to them. Through a part of every day, all the boys that are large enough work in the field, to learn agriculture and form habits of industry; while the girls, under a gifted and devoted matron, practice housekeeping, spinning, weaving, sewing, and the labors of the dairy. Throughout the whole, there reigns as fine an aspect of cheerfulness, obedience, and propriety, as I have ever seen among an equal number of our own children. To this result christianity contributes a full share. Morning and evening devotional exercises are never omitted, and always embrace vocal music, in which the chidren unite; and on the Sabbath the whole attend 'Sunday school,' and afterwards the various exercises of the sanctuary; which, conducted in the fervent and exciting manner of the respectable sect to which the school belongs, are well calculated to rouse the Indian from his constitutional hebetude. Thus, the wants and desires of the whole man—physical, intellectual, and moral, are met by this establishment.

Several advantages are perceived to flow from substituting a central or federal, for the tribe schools. 1st. The children are further removed from the parents who are prone to allure them home. 2d. As they come from different tribes, who speak different languages, they more readily and willingly learn our own as the common medium of intercourse, which thus becomes to them what the Latin once was in Europe. 3d. The presence of a large number increases the excitement and interest of the whole. 4th. The exhibition of several of the useful arts tends to enlarge their minds.

But some of the gentlemen connected with this institution are meditating the re-establishment of the tribe schools, though in a different way from the old. They propose, as soon as practicable, to send back well qualified *Indian* teachers, male and female, to open schools among the tribes to which they belong, and thus relieve the central school from the necessity of receiving very small children. If this idea can be carried out, the effect cannot fail to be excellent.

I ought to state, that in discontinuing the tribe schools, the Methodists have not recalled their missionaries; but only relieved them from the duty of teaching, whereby they are left more at liberty to instruct those among whom they labor, in the precepts and principles of our holy religion, and to assist them, by their example and advice, in rural and domestic economy. In this way I found the Reverend gentleman who resides among the Kickapoos, actually employed a few days since. Another important object is effected by this class of laborers. Mingling constantly with the Indians, and acquiring their confidence, they are able to procure pupils for the central school, who might otherwise never even hear of it.

None of the tribes in this quarter, except the Wyandotts, have even the germ of an organized government, though they are not without certain usages which are perhaps pretty generally observed. The Wyandotts, as you know, lately went from the upper waters of the Sandusky river, in the State of Ohio, and are settled immediately above the junction of the Kanzas with the Missouri river. *They* have an organized government with a body of written laws. The government consists of a president and six counsellors, elected annually, who combine the legislative, judicial, and executive functions. They are as destitute of lawyers as of doctors. In a criminal case, however, the culprit may choose some one to plead his cause. After conviction of murder, the whole proceedings are laid before the tribe for ratification or annulment. The mode of punishment is by shooting. As an example of economy I should state that the annual salary of a member of the Council is $50, that of the President $60. I fancy that if the salaries of our civil officers were modeled somewhat after these, we should not find quite so many of our brethren degrading themselves into politicians and civilians. I ought, in justice, to inform you, that this political system of which my limits will only admit this imperfect notice, was mainly invented by *Sum-men-do-wat*, a full blood and unlettered Wyandott.

As some of our readers may have heard of an extraordinary mortality among this nation last summer and fall, soon after they reached the mouth of the Kanzas, I may refer to it. About 80, out of between 600 and 700 died. They were chiefly children and aged persons, of unmixed blood. The diseases which destroyed them were mainly measles and diarrhœa. They were encamped and greatly exposed on the right bank of the Kanzas river, and lived on a very unhealthy diet. The reason why more Indians than half-breeds died, was because they took less care of themselves. When sick, the Indian and negro are very much alike. Many of the mixed families of this tribe are intelligent and highly respectable.

But I must stop, or you will think I have become as wild as the wilderness in which I roam, and have forgotten that I am writing for a *medical* journal. I will leave the Indians, then, though not the Indian country, till I have given you a page on another topic, which, perchance, may turn out to be a little more professional. But what shall be its title? * * * * * Well! here it comes—rather long, you will say, but I can't make it shorter.

### *Trips into the farther West by Invalids.*

It is well known, that from the bed of the Mississippi to the base of the Rocky Mountains, through an average of 20 deg. of longitude, there is a general ascent, till the western edge of the great inclined plain, in the latitude of 42 to 43 deg., according to Lieut. Fremont, attains the altitude of 7,000 or 8,000 feet above the level of the sea, or about 16 times that of Cincinnati, Louisville, and St. Louis. From this elevation, the mountain range, which divides the waters of the Pacific from those of the Atlantic, rises 4,000 or 5,000 feet higher, having its summits covered with perpetual snow, and consequently condensing the vapors, brought by the west wind from the former ocean. The rivers which meander through this largest of all prairies, are chiefly the Platte, the Kanzas, and Arkansas, in the latitudes of Ohio, Kentucky, and Tennessee. It is mainly along them and their tributaries that any woodlands are to be seen; everywhere else the traveler meets only with such herbaceous plants and grass, as delight in a thrifty soil. At certain times, copious rains fall on this region, but a sandy soil soon imbibes them, and dews are consequently sparing; while permanent swamps and marshes are almost unknown. In fact the air and earth are dry and comparatively free from fogs, musquitoes, and malaria, after we have ascended the inclined plain a few hundred miles from the right bank of the Missouri river.

Such is the outline of the boundless savannahs on which, in these latter days, some enterprising invalids have sought for that health which our polypharmacy had failed to impart.

The gates of entrance into this great field, are the mouth of the Kanzas, in the latitude of Cincinnati, and the mouth of the Platte, about two degrees further north, that is, in the latitude of 41° 02′ 11″. The routes are, along the latter river, in the direction of the South Pass to Oregon, and between the waters of the Kanzas and Arkansas, to Santa Fee and Lower California; there is, also, a road from the valley of the Kanzas, to that of the Platte. The mule is the camel of this desert. Equally adapted to the saddle and the harness, strong, hardy, healthy, not much of an epicure, and capable of subsisting on very little, he may be regarded as the *sine qua non* of the trader, traveler, and invalid in their wanderings over this woodless wilderness. The small mules are preferred to the larger, and females to males. I lately rode one a short distance, which had just brought her owner from California, and found that her sure, easy, and nimble walk, raised in me such an inclination to put off for the Rocky Mountains, that I prudently dismounted, especially as I was not provided with that indispensable implement of the traveler into this newest Far West, a good rifle.

Well, the arrangements are now made for the journey of health, but who are the invalids that may undertake it, with the hope of benefit? I answer: First. *Dyspeptics*, who by intemperance, gormandizing, idleness, or hard study have broken down their digestive organs. Second. *Hypochondriacs:* such as students of medicine who have been rejected after a third examination—unfortunate speculators, who are unwilling to work, and want time for meditation on future schemes—disappointed office hunters, who have cracked their voices in proclaiming their merits to the people, and dismissed office holders, who would otherwise make a 'voyage up Salt River.' Third. Those who labor under chronic hepatitis and jaundice. Fourth. *Consumptives*, who are not too far advanced to take the saddle.

Let us now inquire for a moment into the circumstances from which the proposed benefits will result. 1st. Extrication from the luxuries and dissipations of society. 2d. The regular and long-continued exercise. 3d. The reduced and simple diet. 4th. Lodging on the ground, amidst the short grass. 5th. The constant immersion of the body in a dry unmalarious, and invigorating atmosphere. 6th. The exercise of the senses on new objects and scenes. 7th. The excitement connected with the danger of being lacerated by the bullets of the *Pawnees;* pierced to death by the arrows of the *Blackfeet*, or picked to death by the *Crows.*

Possessing these active qualities, a trip to the Rocky Mountains cannot fail, in numerous cases, to accomplish more than every other remedy, and I beg leave to commend it to all who are interested, as a measure that will either kill or cure.

### *The Inundation.*

Having seen the great flood, or its desolating effects, from New Orleans to Fort Leavenworth, a distance of 1600 miles, I should be somewhat prepared to speak of it, if my notes were not at St. Louis. Howbeit, I will send you a few memorandums from memory that our journal may not be altogether without a record of this memorable event.

When I left New Orleans, on the 18th of May, the Mississippi was high and still rising, chiefly from the waters of Red River and the Arkansas; both of which had been swollen far above their usual elevation; destroying many plantations, which had never before suffered from inundation. As I slowly ascended the great river through the month of June, it was still rising, for then the waters from the upper rivers were coming down in unwonted volumes. In some places, new levees had given way, and the cotton fields were deluged, as the currents sought an outlet through the Tensas and Washita into Red River, which had then partially emptied itself. At Vicksburg on the 4th of July, the surface of the river had been for about a week at the same height. In ascending the Yazoo, which enters the Mississippi 12 miles above that city, the river was only known by the winding vista through the groves of cotton wood, for the waters spread off indefinitely on both sides, except where the channel happened to lie near the tertiary bluffs to the south. Before reaching the mouth of the Arkansas, we observed that the flood had begun to subside, but were never out of sight of lateral currents, flowing off more copiously to the west than the east. In general they were not so deep as to wash away the fences; but exerted on the corn and cotton a pernicious influence, and subjected the inhabitants to great loss and inconvenience. Above New Madrid, for one stretch of 15 or 20 miles, there was an uninterrupted current departing for the lower grounds, the bayous, lagoons, and lakes, through which the St. Francis makes its way, nearly parallel to the Mississippi, into the Arkansas. On reaching the mouth of the Ohio, on the 11th or 12th, we found the subsidence still more conspicuous. From thence to St. Louis, the drowned lands were reappearing, but in many places the waters were still flowing over banks which they rarely surmount. The desolations on this portion of the river were most melancholy, and indicated a higher flood than what had occurred below the mouth of the Ohio. All the fences were, in whole or in part, swept away; small framed houses, and the framed roofs of log houses, were lodged among the cotton trees, larger houses still had currents running through them, and some were tied to trees with ropes, to prevent their floating off. The inhabitants had all fled; and the only domestic animals, which I saw on any alluvial plantation. for 200 miles, were two flocks of geese! Even the leaves of many of the forest trees and shrubs, (the species 1 noted at the time, but cannot recount them from memory), had changed to a yellow hue and begun to fall. The cotton tree was alone seen to be green and growing in the midst of this 'watery waste.' In ascending the Missouri to Fort Leavenworth, about 400 miles, a scene of still greater devastation met the eye. For the torrent had been more copious and rapid, the drifting sands more abundant, and the quantity of floating timber much greater. By these agencies every plantation on the river bottoms was nearly destroyed. In many places, large slices of bank with their houses and barns were cut away, the fences entirely carried off, and almost every remaining vestige of art buried up beneath sand and wood. Of course this avalanche of drift generally rests near the margin of the banks, though in many instances it is spread over the entire plantation, which is thus reduced to utter sterility. Such is the aspect of either bank of the Missouri as far as I ascended that river. Of the condition of those of the upper Mississippi I can say nothing from personal observation. It is affirmed by many persons that this is the greatest flood which has appeared in these latitudes since the year 1785. Should it not be visited by another for the next sixty years, that long period would not be sufficient to restore some plantations to their late fertility.

It will be inferred from what has been said. that the Lower Mississippi did not rise as high as the middle, and such was the fact. Nor did the Upper Missouri experience the same swell as the portion of which I have spoken. From the best accounts I have been able to obtain, there was no remarkable rise above the Platte, which, flowing nearly east, enters the Missouri 300 miles above Fort Leavenworth; and even above that post, the rise was less than below. Thus it was not the descent of melted snows through the Upper Missouri and its great tributary, the Yellow Stone, but the fall of rains in a region south of the sources of the latter, that deluged the vallies of Red River, the Arkansas, Kanzas, and the Little Platte, and the great Osage of the State of Missouri. While this was going forward, Louisiana, Alabama, Mississippi, Tennessee, and Kentucky appear in no degree to have suffered. In fact, throughout the months of April and May the maritime portions of the three former, actually suffered from drought, to such a degree, that in Louisiana, the sugar planters on the first of June were irrigating their plantations, by cutting trenches through the levees, that the waters of the swollen Mississippi might escape upon them. On the 14th of that month, the flood in the Missouri, opposite where I now am, was at its height, showing, conclusively, that great rains had fallen to the west, while in the south there had been none. It should be borne in mind, however, that June is the time for the annual rise of the Upper Mississippi and Missouri, which rivers were, therefore, in a state of repletion, at the time when the western tributaries of these middle latitudes, threw out their desolating torrents. Deprived of the opportunity of seeing newspapers, I am not informed as to the extent of this rainy zone to the east, but have understood that the Illinois rose to a great height, and if so, the region of excessive rain, extended, perhaps, from the Rocky Mountains to Lake Erie.

Will the time ever come, when the Missouri and Mississippi will no longer overflow their banks? When, between deposits made upon them and the excavation of their channels, the latter will become sufficiently capacious to contain all the waters which may press into them? This question, I think, must be answered in the negative. It is true, that every flood raises their banks and bottom lands, but it also raises their beds. And this must be the case with all alluvial streams, for they continually bring down and deposit sand and other drift in their channels. Thus it is, that the valleys fill up, while the channels of the rivers flowing along them, retain the same capacity. The beds rise with the banks, and the whole stream comes to occupy a higher level.

There is a great difference, in its effect on the fertility of the soil, between an inundation by the Missouri, and one by the Lower Mississippi; for as sand is borne along with difficulty, it is deposited above, while clay, marl, and comminuted plants are carried on and made to fertilize the more distant regions.

Will the late inundation be followed by great autumnal sickness? Time will tell us. Meanwhile I may remark, that among the scattered population about the mouth of the Kanzas, there are at this date, August 7th, more than 20 cases of intermittent fever, generally, however, of a mild character. I might say more on this subject, but, unwilling to inundate you with ink, will forbear.

And yet I cannot close, without touching on some other matters. I had hoped, that I might find on the banks of the Kanzas, some plants that would be new to our respected friend and colleague, Professor Short; but am disappointed. Identity of latitude, soil, and elevation, gives such perfect identity of vegetation, at a distance of 1,000 miles, as would greatly strengthen our venerable friend, Professor Caldwell, if he could witness it, in his opinions on spontaneous production.

A word on Ornithology, and I am done. In days long gone by, the *Paroquet* enlivened the embowering forests of the banks of the Ohio, as far up as Cincinnati; but its green plumage no longer emulates the foliage of the woods. Nor did I last spring in the south-west, nor this year in that region, or in ascending to the Mississippi, once hear its unmelodious note. Here, however, it has greeted me many times, and flocks are frequently seen flitting among the green trees. Thus, like the Indian, this native bird is retiring to the west, and with him, melancholy thought! may at last become extinct. To this retirement, the noisy and smoking steamboat has no doubt contributed a full share; as the Paroquet delights to dwell in the neighborhood of quiet and shady valleys. With this *feathering* out, I shall conclude, by subscribing myself

Your friend and colleague,

